# Parasuicidal behavior in early stages of psychosis

**DOI:** 10.1192/j.eurpsy.2023.2225

**Published:** 2023-07-19

**Authors:** B. Serván Rendón-Luna, A. Montes Montero, P. Gómez Merino

**Affiliations:** Hospital Clínico San Carlos, Madrid, Spain

## Abstract

**Introduction:**

Psychotic experiences (PE) are strongly associated with non-suicidal self-harm (NSA). NSA are present throughout life, but are more frequent during adolescence and young adulthood. Early psychotic episodes (PEP) are a particularly vulnerable group compared to later phases of psychosis psychosis.

**Objectives:**

Analyze risk factors for suicide attempts and NSA, in order to improve early detection and prevention of suicides in adolescents and young adults with PD

**Methods:**

Review in the literature of the different risk factors associated with parasuicidal behaviors in early psychosis

**Results:**

- Presence of positive psychotic symptoms: auditory hallucinations, Delusional ideation.
- Social isolation
- Longer duration of untreated psychosis.
- Comorbid symptoms: irritability, depression, anxiety, psychotic distress, insomnia.
- Traumatic events in childhood
- Difficulty in regulating emotional, impulsivity and sensitivity to reward.
- Consumption of substances.
- Psychosocial stress.

**Conclusions:**

We consider essential the inclusion of early intervention programs aimed at the prevention of suicide and NSA, evaluating all risk factors for suicide and NSA among individuals with a PEP and high-risk mental states.

Initial assessment and ongoing assessments of suicide risk and parasuicidal behaviors, positive psychotic symptoms, depression, and the other related risk factors mentioned are required. Integrating trauma management into PEP care is critical.

**Bibliography:**

1.- Honings S, et al. Psychotic experiences and risk of self-injurious behaviour in the general population: a systematic review and meta-analysis. Psychol Med. 2016;46(02):237–51. doi: 10.1017/S0033291715001841

2.- Honings S, et al. Psychotic experiences and incident suicidal ideation and behaviour: Disentangling the longitudinal associations from connected psychopathology. Psychiatry Res. 2016;245:267–75. doi: 10.1016/j.psychres.2016.08.002

3.- Nishida A, et al. Risk for suicidal problems in poor-help-seeking adolescents with psychotic-like experiences: Findings from a cross-sectional survey of 16,131 adolescents. Schizphr Res. 2014;159(2–3):257–62. doi: 10.1016/j.schres.2014.09.030 39.

**Disclosure of Interest:**

None Declared

